# A narrative review: suicide and suicidal behaviour in older adults

**DOI:** 10.3389/fpsyt.2024.1395462

**Published:** 2024-05-10

**Authors:** Joseph Sadek, Bryan Diaz-Piedra, Leah Saleh, Luke MacDonald

**Affiliations:** ^1^ Professor, Department of Psychiatry, Dalhousie University, Halifax, NS, Canada; ^2^ Faculty of Science, Dalhousie University, Halifax, NS, Canada; ^3^ MD Candidate, Faculty of Medicine, Dalhousie University, Halifax, NS, Canada

**Keywords:** elderly/geriatric suicide, self-harm, risk factor, prevention, screening, assessment

## Abstract

Globally, suicide is a public health concern that claims the lives of many each year. The complex etiology and factors contributing to the risk of suicide make it hard to predict the likelihood of death by suicide. Suicide rates have been increasing over the past 25 years in patients aged 65 years and older, and with the expected increases in the size of the older adult population and the under-detection of suicide risk, these rates may continue to increase. To mitigate and attempt to limit this expected increase, it is important to understand the risk and protective factors of suicide in older adults. This narrative review focuses on individuals above the age of 65 and encompasses relevant peer-reviewed publications from the past 25 years to cover fatal and non-fatal suicidal behaviour. It summarizes several important risk factors for suicide and suicidal behaviors while considering how risk can be detected, assessed, prevented, and mitigated. Screening methods to detect suicide and depression in older adults were examined based on their effectiveness and suitability for use in this population. Lastly, the impacts of the COVID-19 pandemic on suicide rates in older adults were described.

## Introduction

The World Health Organization states that suicide is a global public health concern, accounting for approximately 700,000 deaths per year. The majority of completed suicides occur in low-and-middle-income countries (77%), which accounts for 84% of the world’s total population (1). Globally, suicide mortality rates have been decreasing over the past 20 years (2), however, the rates of suicide in adults aged 60 or older in the Americas have been gradually increasing (3). Mental health disorders are a major risk factor that contributes to these increasing rates. With disorders such as depression becoming more prevalent in the Americas, some populations are more at risk than others. Older adults who are depressed are more likely than those who are not depressed to harm themselves or attempt suicide (4). Old age is characterized by losses in physical and cognitive functioning, self-determination, and increases in social isolation. The process of aging is often associated with a decrease in physical and functional capabilities, however, one’s age may not coincide with their abilities and may more accurately be considered a social construct (5). The World Health Organization defines old age as commencing when one becomes eligible for retirement and various medical-related benefits, which occurs upon finishing your 60^th^ year of life (6). The inability to depict a universal elderly age may be attributable to varying socio-demographic and quality-of-life-related factors (7). Such factors exacerbate feelings of anxiety and helplessness in older adults and are strongly correlated with depression arising from a perceived decline in quality of life (8). Non-suicidal self-injury (NSSI) refers to behaviors with the intent of harming oneself without the intention of killing oneself such as cutting or self-harming. Patients who had suicide attempts may have thought of permanent relief by death.

## Methods

The literature review was conducted in the following databases: Medline/Pubmed, Google Scholar and PsycINFO. Search terms used were suicide and elderly; older adults; suicide attempts and elderly; suicidal behaviour and elderly; COVID-19 and suicide; geriatric suicide; suicide prevention and elderly; geriatric suicide; older adults and suicide; older adults and self-harm; geriatric self-harm; and risk factors.

Publication dates were restricted to a period from January 1, 1998, to October 1, 2023, and the language was filtered to include articles written in English.

Following the literature search, identified articles were subjected to a screening process. Initially, duplicate records were removed. Subsequently, two independent reviewers (science students) manually screened the titles and abstracts of the remaining articles. Full-text peer-reviewed articles deemed potentially relevant based on the screening of abstracts were retrieved for further assessment. Qualitative studies were not included in the search and selection process.

## Results

### Suicide epidemiology in older adults

The number of deaths caused by suicide in older adults has been steadily increasing over the past 19 years in the United States. In 2001, the population was 285,470,493 and suicide accounted for the death of 6,725 adults aged 60 or older leading to a rate of 2.3 in 100,000. Since then, this number has greatly increased to 12,470 deaths by suicide in 2020 with a population of 335,942,003 with a rate of rate of 3.7 in 100,000 (9). The rates are roughly getting close to doubling over 19 years and are likely to continue growing if no actions are taken. **Table 1** shows the increase in the number of deaths caused by suicide for adults aged 60 or older. Due to the increasing number of deaths and the lethality of old age suicide, it is important to focus on older adults.

**Table 1:** Number of Older Adult Suicides in the United States.

Year	Deaths by Suicide
2001	6,725
2002	6,980
2003	6,777
2004	6,860
2005	7,137
2006	7,090
2007	7,528
2008	8,008
2009	8,175
2010	8,618
2011	9,034
2012	9,492
2013	10,189
2014	10,935
2015	11,193
2016	11,588
2017	12,131
2018	12,890
2019	12,839
2020	12,470

Number of deaths by suicide in the United States from 2001 to 2020 in adults older than 60 from the WISQARS Injury Mortality Report Visualization Tool (9).

Currently, there are roughly 1 billion people aged 60 or older in the global population. As the baby boom generation continues to age, this number is expected to increase to about 1.4 billion by the year 2030 (6). This growth in the geriatric population means that the deaths caused by suicide in the geriatric population could also increase drastically. Older males have higher rates of death by suicide than older females. Common methods of suicide include hanging, using firearms, jumping from high places, overdoses, and drowning. Suicide rates may vary depending on the geographical location (10). Rates of elderly suicide were reported to be 18–22 per 100,000 older men and 3.5 to 4.5 in 100,000 older females.

To combat the expected increase in geriatric suicides, it is necessary that instruments to both assess and manage suicide are developed and implemented in clinics and emergency settings. Moreover, due to the fatality of suicides in older adults compared to younger adults, more attention should be directed toward these populations (11). The need for additional attention is especially important because it has been estimated that by the year 2050, there are going to be about 2.1 billion adults aged 60 years or older, which is close to double the current geriatric population (6). Regarding the prevalence of suicidal ideation in older adults, a study by Fässberg et al. (12) provides insight into the epidemiology of suicidal feelings in an aging Swedish population, ranging from old to very old age in the Gothenburg H70 Birth Cohort Studies. This study sheds light on the frequency and nuances of suicidal ideation in older individuals, contributing valuable data to our understanding of this critical issue. The lifetime prevalence of suicidal feelings was 25.2% and prevalence rates increased with age in older females but not older males.

### Overview of risk factors for suicide in older adults

Various categories of risk factors can increase the probability that older adults may attempt suicide. Reasons for attempted suicide in late life include “desire to escape, reduced functioning and autonomy, psychiatric disorders, somatic problems, and physical pain, perceived burdensomeness, social problems, family, conflict, lack of meaning in life, and some patients had no specific reason” (13). Limited social connectedness is associated with suicidal ideation, non-fatal suicidal behaviour, and suicide in later life (14). Despite older adults being at significant risk for suicide, they rarely receive the awareness and harm reduction interventions necessary to be preventative in nature (15). Rather, feelings of depression stemming from loneliness and isolation have become normalized in the aging population, facilitating a significant increase in suicide among these individuals (16). The common risk factors that make the geriatric population susceptible to suicidal behaviour consist of social isolation, existing mental health implications with special emphasis on major depressive disorder, suicidal ideation and previous suicide attempts, geographical location, socioeconomic status, drug use, physical health problems such as cancer, and the normalization of ageism in modern society.

Social isolation has been a longstanding concern among individuals in the geriatric population and COVID-19 restrictions within recent years have only exacerbated this effect, significantly impacting the quality of life of older adults (17). With higher rates of suicides, and increased likelihood of death from suicide in this population, there are specific implications that need to be considered. Loss or degradation of interpersonal relationships through a variety of means, especially loss of a spouse, is a major contributor to social isolation in the geriatric population (18). Not only are spouses an enormous social support themselves, but individuals are frequently connected to social communities and other interpersonal relations through their spouses, which can be majorly affected following death or loss (17).

When it comes to why suicidal ideation is a frequent occurrence in older adults, social isolation is only one of many contributors. An exploratory study published in the Canadian Journal of Aging found that thoughts of suicide in elderly individuals are a means of alleviating psychological suffering that occurs because of lost loved ones and other stressors (19). They also identified three basic needs that affect the tendency of some older adults to think about self-harm and suicide: the need to self-actualize, the need to belong, and the need to feel safe. Failure to meet any of these goals is a significant contributing factor to thoughts of suicide experienced by the aging population (19).

Depression has long been associated with an increased risk of suicidal thoughts, and suicide attempts, and the presence of depression in geriatric patients is no exception (20). There are some affective symptoms, however, there are unique findings in patients of advanced age, one of the most prominent being perceived burdensomeness (21). Perceived burdensomeness refers to a perception that one is a burden or drain on their loved ones, and this is a feeling that plagues many older adults who rely on their spouses, children, and other significant others for certain aspects of living (21). One small study with 106 older adults in the primary care setting found that this affective symptom of perceived burdensomeness accounted for 68.3% of the variance in suicidal ideation experienced by older adults at risk of depression (22).

### Psychiatric disorders

Major depressive disorder (MDD) is an increasingly prevalent disorder that has been projected by the World Health Organization to become the leading global burden of disease by the year 2030 (23). Moreover, in adults aged 18 years and older living in Canada as of 2021, 15.2% have screened positive for MDD (24). Given the overwhelming presence of major depression today, it is worthwhile to understand the criteria that must be met to achieve a diagnosis of this debilitating disorder. MDD has a biopsychosocial etiology. It is found to be heavily influenced by external factors, such as environmental, and psychosocial factors in addition to internal genetic and biological factors (25). MDD is characterized by at least 2 weeks of consistently low or irritable mood, a decline in interest in previously gratifying activities, reduced energy, feelings of worthlessness, poor concentration, irritability, appetitive changes, suicidal ideation and behaviors, and disrupted sleep presenting either as insomnia or oversleeping To date, MDD is diagnosed based on the diagnostic criteria outlined in the current version of the DSM-5 (26).

MDD is an imperative indicator of suicide in the geriatric population (8). MDD has been depicted as the most prevalent psychiatric diagnosis among old age individuals who die from suicide and there is a significant positive correlation between the presence of MDD and suicide attempts among elderly members of society (8). Comprehension of the concurrency of these two factors is critical for early intervention and prevention of suicide among older individuals. Recent research highlights the importance of considering the implications of cognitive impairment and depression on the progression of dementia. Seeing as depressive disorders and dementia are two of the most common diagnoses made among the geriatric population, exploring how depression and its wide range of symptoms play a role as a risk factor for various types of dementia is a vital domain of research. The presence of symptoms of depression is frequently detected in the early stages of asymptomatic Alzheimer’s disease, or AD (27). The stage of neurological decline that precedes dementia is referred to as mild cognitive impairment, or MCI, and has been found to be concurrent with depression in old age individuals (28). Moreover, the incidence of depression in late life is a vital predictor of the progression of MCI in otherwise healthy individuals (29).

Patients diagnosed with dementia generally have an elevated risk of suicide (30), as recent research suggests that the risk of suicide increases within one year after the diagnosis of dementia (31). A Swedish national-based study suggested that suicidal behaviour increases in patients who had previous self-harm, had serious depression, used anxiolytics or hypnotics, and those who have milder dementia and higher frailty scores (32). To assess the relationship between depression and the progression of dementia, amyloid beta peptides and tau proteins are Alzheimer’s biomarkers that have been designated as predictors with research depicting that increased quantities of these biomarkers may be associated with depression (33). Moulinet et al. conducted a study to analyze the association between depression and Alzheimer’s biomarkers across both preclinical and clinical stages of the disease in older adults (2022). The participants varied in that a subset were healthy controls. (HC), some presented with subjective cognitive decline (SCD) with no probable AD, and the remainder were older adults on the Alzheimer’s continuum (ADC) with probable AD. The results of this study depicted that depressive symptoms were higher in all patients with SCD and ADC compared to HC. Moreover, depressive symptoms were correlated with greater quantities of amyloid-beta in the SCD group, but not in the HC or ADC group (34). These results reveal the relationship existing between Alzheimer’s biomarkers and depression in patients with pre-existing cognitive decline (34).

Some older adults with MDD may experience a variety of different cognitive deficits. These deficits occur in different neuropsychological domains, such as working memory, executive functioning, visual and verbal memory, cognitive control, and attention (35). Specifically, deficiencies in cognitive control can increase the risk of suicide in geriatric patients with late-life depression (35–37). Cognitive control refers to the ability to coordinate and regulate any thoughts or actions that are in line with behavioural goals (38). Patients with MDD might experience declines in their cognitive control, subsequently hindering their ability to regulate their suicidal thoughts or intentions and therefore causing increases in the risk of suicide (37). Additionally, these age-related declines reduce the ability of older adults to adapt and respond to stressors, increasing the likelihood of them seeing suicide as the only feasible solution (36, 39). Pu et al. used the Brief Assessment of Cognition in Schizophrenia (BACS) to examine the six cognitive domains (working memory, verbal memory, motor speed, verbal fluency, attention, and speed of information processing) of their outpatient study participants (2017). They determined that there was a negative correlation between executive functioning, motor speed, composite scores, and suicidal ideation. This relationship indicates that decreases in these cognitive domains are associated with increases in suicidal ideation and potentially even suicide (35). Another study used the Montreal Cognitive Assessment (MoCA) to assess the cognitive integrity of geriatric patients with late-life depression. Their results revealed that poorer performance on the MoCA was associated with suicidal ideation in geriatric patients (37). Cognitive assessments should not replace suicide screening methods but should be used as a tool to identify lower cognitive scores before thoroughly assessing suicidal behaviors.

Other psychiatric disorders that are associated with suicide include schizophrenia, bipolar disorder, alcohol, and substance use disorders (10).

### Substance use

The baby boom generation has significantly higher rates of marijuana and illicit drug use when compared to other generations (40). As this generation continues to age, drug use in older adults is expected to increase, which poses a risk to the physical and mental well-being of people in this age group. Moreover, due to physiological changes associated with aging and diseases, drug pharmacokinetics may be affected, leading to longer-lasting and greater drug serum levels in the body (41). These changes can also increase the risk of there being adverse drug reactions (41). Marijuana is typically considered to be more therapeutic and pleasurable rather than harmful in the geriatric population (42). As the THC content of cannabis preparations increases, the adverse effects in older adults also increase. Some of these risks include worsening cognitive decline and medical conditions, interacting with other medications, and increasing the risk of falls (42). Additionally, the use of marijuana increases the risk of major depressive episodes (MDE) and suicidal ideation. The risk of having either MDE or suicidal ideation was linearly associated with marijuana use frequency, meaning that more frequent use of marijuana increases suicidal risk (42). Meanwhile, using marijuana in conjunction with other illicit drugs significantly increased the odds of both MDE and suicidal thoughts. Substance use may also increase the risk of suicide through cognitive effects such  as increased impulsivity, and decreased inhibitory control. Consequently, primary care physicians need to screen for drug use in older adults to identify risks and provide acceptable treatment. The relationship between marijuana use and suicidal behaviors is understudied and would benefit from additional research.

In psychological autopsy studies set in Western countries, alcohol use disorder is the second most common diagnosis among older adults who die by suicide. Alcohol use disorder increases the risk of suicide in both older men and older women (43). In a study of older hospitalized patients after suicide attempts, half of the patients who had the diagnosis of alcohol use disorder had at least one prior suicide attempt versus one-third who did not have alcohol use disorder (44). Within older adults, alcohol consumption has been increasing because adults have been living longer and healthier lives (45). For those aged 65 or older, binge drinking has been increasing by about 3.4% per decade, which has resulted in alcohol use disorders also rapidly increasing (46). Alcohol generally has greater negative effects on older adults because of physiological changes that occur with aging (47). These changes result in alcohol being metabolized more slowly, leading to higher blood alcohol levels with the same or lower levels of consumption (46, 47). Older adults who are high- or low-alcohol consumers have an elevated risk of experiencing depressive episodes. However, moderate consumers had a lower risk of depressive episodes (46). The lower risk of depressive episodes in moderate consumers may be caused by the stress-response-dampening effects of alcohol (48). Regardless of these dose-dependent effects, all quantities of alcohol had some effect on the risk of depressive episodes, and indirectly suicide risk. With the older adult population increasing and the rising use of alcohol, this poses a significant risk to the prevalence of suicide attempts in this age group. Consumers should be regularly questioned regarding their alcohol consumption to reduce the risk of depressive episodes and suicide attempts.

In hand with the increasing population of older adults, there is an increase in the number of them requiring long-term care. Drugs and medications often have greater adverse effects on these populations because of their greater frailty, medical comorbidities, and greater degrees of polypharmacy (49). Drugs such as antidepressants and psychoactive medications are frequently used in long-term care facilities. For those prescribed antidepressants, there is a decreased risk of suicide. However, long-term care patients who were prescribed hypnotics, a commonly administered drug, had twice the risk of suicide (50). The increased risk may be associated with the drug’s ability to worsen judgement, depression, and cause behavioural confusion. Additionally, the hypnotics may impair normal cognitive functioning (51) leading to pharmacological overdose (52). Pharmacological overdoses are not overly common in older adults as prescription drug misuse (PDM) is less likely in those who are 65 or older compared to those who are 50–64 (52). Regarding PDM, there is an inverse relationship between PDM and age, such that as aging occurs, the likelihood of PDM decreases. This relationship could be caused by the elevated risk of adverse health effects, which can produce a physiological signal indicating that drug use needs to be reduced (52).

### Medical illness & social factors

In examining the complexities of late-life suicide, it is imperative to consider the interplay between physical illness, functional disability, and suicidal behaviour among older adults. Research by Fässberg et al. (53) emphasizes the significance of this relationship, highlighting how physical illness and functional limitations can significantly contribute to suicidal ideation and behaviour in older individuals. Chronic health conditions, mobility impairments, and limitations in activities of daily living can exacerbate feelings of hopelessness and despair, thereby increasing vulnerability to suicide. Furthermore, functional disability may act as a barrier to seeking and receiving appropriate mental health care, further compounding the risk of suicidal behaviour among older adults. Suicidal behaviour, maybe associated with functional decline and some medical problems, such as malignancy, chronic pain, COPD, liver and kidney disease, male genital disorders, neurological disorders, and arthritis. (53).

In addition to physical health factors, social determinants play a crucial role in late-life suicide risk. Bereavement, in particular, has been identified as a significant risk factor for suicidal behaviour among older adults. Erlangsen et al. (54) conducted a population-based register study highlighting the heightened suicide risk among the oldest old following the loss of a partner. This underscores the profound impact of social losses on mental well-being and emphasizes the importance of targeted interventions to support bereaved individuals in coping with their grief and reducing suicide risk.

Furthermore, social factors beyond bereavement contribute to the complexity of late-life suicide. Fässberg et al. (14) conducted a systematic review exploring the relationship between various social factors and suicidal behaviour in older adulthood. Their findings underscored the significance of social support, social isolation, and interpersonal relationships in influencing suicide risk among older individuals. Addressing social determinants of health, such as enhancing social connectedness and reducing social isolation, is essential in comprehensive suicide prevention efforts targeting the geriatric population.

Moreover, personality traits (particularly Borderline and other cluster B personality disorders) and characteristics have garnered attention in understanding late-life suicide and suicide attempts. Studies have explored the influence of personality factors such as neuroticism, impulsivity, and resilience on suicidal behaviour among older adults. Understanding the interplay between personality traits and other risk factors can provide valuable insights into tailored interventions and strategies for suicide prevention in this vulnerable population.

In summary, a comprehensive understanding of late-life suicide necessitates an exploration of physical health, social determinants, and personality factors. By addressing these multifaceted dimensions, clinicians and researchers can develop targeted interventions and strategies to mitigate suicide risk and promote mental well-being among older adults.

### Impacts of COVID-19 on suicide rates

In 2020, the World Health Organization declared COVID-19 a pandemic, which changed the lives of many and resulted in approximately 7,000,000 deaths (55). With increased social isolation due to strict isolation procedures, feelings of depression and anxiety were common in the general population (56). Several studies predicted that the suicide rates in older adults would increase with the onset of COVID-19 (57–59). These predictions were based on a variety of factors that would increase the risk of suicide attempts in members of the older population. Some of these risk factors included social disconnection (59, 60), thwarted belongingness (59, 61), and perceived burdensomeness (59, 62). These factors increased feelings of loneliness and led to increased levels of social isolation (59, 60), which added to the already pre-existing levels of loneliness they typically experienced. In addition to these factors, their self-worth and value in society were often diminished due to the way the media talked about older adults. Members of the older population would frequently hear how they are less of a priority than younger people (63). Various trends like “#BoomerRemover” would exacerbate the effects of ageism and worsen and undermine their self-worth (64).

As new articles are published, there are contrasting views in terms of the changes in suicide rates after COVID-19 in older adults. Some literature reports that COVID-19 has increased both self-harm (65) and suicide rates in the older population (59, 61, 62, 66). Self-harm is considered an important risk factor for suicide, as there is a 67-fold increase in the chance of dying by suicide for older adults with a history of self-harm (65). Increased periods of social isolation are thought to increase the likelihood of self-harm; however, this relationship is largely understudied and could benefit from more extensive research (65). The relationship between suicide rates and COVID-19 is complex in nature, but many articles refer to the Interpersonal Theory of Suicide (ITS) to help explain the increased desire and risk for suicide. In the ITS model, the desire for suicide stems from their thwarted belongingness and perceived burdensomeness, which often arise when people experience social isolation, lack of social support, loneliness, and functional impairment (67). The strict and highly enforced isolation procedures during the pandemic increased these factors in many older adults, which subsequently increased their desire and risk for suicide (59, 68).

A few studies have stated the opposite, describing how there were no significant differences in depression (60, 69) and suicidal ideation after COVID-19 (60). These studies describe how older adults have greater resiliency and are better able to adapt to stressors and overcome challenging situations. In addition to resiliency, the isolation procedures lead to older adults communicating more frequently with their family members, which increases their perceived social support (60). This increase in perceived social support is a protective factor, that acts to mitigate some of the risks associated with isolation and depression. Another protective factor for suicide in older adults is religion and spirituality (R/S) (62, 70). It is believed that R/S improves subjective well-being (60) and strengthens religious beliefs about suicide behaviors during times of crisis (70). Even with these protective factors, it is recommended that additional studies are completed to confirm these relationships.

When considering gender differences, Kim et al. (66) describe how suicide rates significantly increased in adolescent males, and adult and older adult females. In women aged 65 and older, suicide rates increased by 12.5% after COVID-19. A factor that could contribute to these elevated rates is the general increase in impulsive suicides, which increased in all age groups after the pandemic (66). Another potential explanation could be how women were more likely to be negatively impacted by COVID-19, resulting in a greater chance of developing emotional distress symptoms (69). Lastly, an important consideration is that older women are less likely to live alone compared to older men (69). These findings would suggest that men would be more likely to experience social isolation and subsequent emotional distress, but these were not the noted findings, indicating the need for further research.

### Previous suicide attempts

When discussing old age in adults, the age categories can be further subdivided into young-old and old-old. The young-old age group spans from 65–74, while the old-old age group is those who are 75 or older (71). Within these age groups, the effects of previous suicide attempts have different levels of risk. Previous suicide attempts pose a greater risk of recurrent suicide attempts for the young-old geriatric population. In this age group, not all adults are diagnosed with conditions or diseases that deteriorate their physical well-being and mental health. Suicide attempts are less likely to be fatal on their first attempts, and consequently increase the risk of another suicide attempt (39, 71). For the old-old age group, their physical frailty and vulnerability due to their older age results in most suicide attempts being fatal (72, 73). Moreover, these age groups more carefully plan their attempts (74) and choose methods that are known to be more lethal and fatal at completing suicides (75). As a result, it is unlikely that there will be previous suicide attempts that could increase the risk for subsequent attempts (71, 76). Comparing the older age group (65+) to the younger age group (<65), the older age group is 3 times less likely to have previous suicide attempts when compared to the younger age group. Despite this 3-fold difference, previous suicide attempts still increase the risk for subsequent attempts in older adults (39).

### Strategies for mitigating suicidality

Despite the incidence rate of suicide among older adults, the research and implementation of preventative measures are severely neglected (77). Research suggests that suicide in older adults is marked by high intentionality and increased fatality (11). This makes sense given their frailty, tendency to meticulously plan (74), and lower likelihood of interruption which puts them at an increased risk for death from suicide attempts (9). With these factors in mind, implementing protective and preventative measures in society is critical to mitigate the fatal outcomes of suicide in older adults, and subsequently utilize early interventions to prevent attempts from initially occurring. Suicidality in older adults encompasses 20% of all global suicides, with the fatality rate attributable to geriatric suicide being 150,000 per year (78). A key determinant to identifying suicidal individuals is to assess the extent of suicidal ideation they possess, with suicidal ideation manifesting as a range of emotions from passive thoughts of death to premeditated detailed plans on how one wishes to perish (79). Suicidal ideation is often dismissed in older adults and depicted as a typical side effect of aging. Our modern ageist society facilitates the increasing geriatric suicide rates by allowing thoughts of death and depression to be normalized and subsequently undetected, furthering the social isolation that older adults are prone to feeling.

Consequently, suicidality in older adults can be mitigated by implementing preventative measures in four domains (79). The first domain consists of integrating intensive screening protocols in primary care settings, seeing as most suicidal elders see their primary care physician the year before attempting suicide. Depression screening and management in primary care settings particularly in collaborative care had the strongest evidence (80). The second requires modifying society’s approach to aging, reducing ageism and combatting the normalization of depression among older adults. The third relies on furthering existing research on older adults’ suicide and how symptoms present in this population, mainly so that common warning signs can be made aware to the public and health care workers. Finally, the fourth entails implementing accessible supportive resources for older adults, such that they do not feel isolated and have outlets to discuss their feelings rather than having them dismissed (79). Early screening and intervention are vital to identifying at-risk individuals and providing them with the necessary resources to cope, rather than resorting to harmful coping mechanisms. Such resources include but are not limited to cognitive behavioural therapies (CBT), interpersonal therapy (IPT), supportive psychotherapy, problem-solving therapy (PST), involving family member/caregiver/environment in therapy (Ecosystem focus therapy), screening mechanisms such as the GDS and DIA-S, pharmacotherapy and antidepressant treatment, community-based outreach programs, counselling in person or via telephone for vulnerable adults, educating primary care physicians on screening protocols and early detection, and the combined effects of exercise and medications on reducing suicidality (80, 81).

### Medications and exercise

Researchers often question the use of antidepressants in older adults due to the adverse effects that some people experience, such as hyponatremia, gastrointestinal bleeding, and negative interactions with other drugs (82). Not only that, but the effects of antidepressants on people in the older adult population have differing effects depending on the type of antidepressant administered and the number of antidepressant treatments they are on. Additionally, the effects of antidepressants may also differ due to differences in pharmacokinetics due to aging (83). Hedna et al. describe how older adult individuals on a single antidepressant treatment of mirtazapine or those taking more than one antidepressant at a time had an increased risk of suicidal behaviour compared to those on a single selective serotonin reuptake inhibitor (SSRI) treatment (84). However, it is important to consider that those individuals who were on mirtazapine or taking more than one antidepressant were also more likely to use specialized healthcare systems to treat their depression. Individuals who are taking more than one antidepressant may require specialized services since they may be refractory to treatment or have a severe form of depression that subsequently increases their suicidal risk. Some other antidepressants that were noted to increase suicidal behaviors were fluvoxamine and venlafaxine (84). These effects were attributed to their underlying mechanisms which had subsequent impacts on concomitant drug administration and increased the risk of adverse effects and hospitalizations (85).

The study by Hedna et al. outlined how two-thirds of the older adults who died by suicide were not on antidepressant treatment while the other one-third had filed a prescription within the last three months of their life (2021). This evidence highlights the lower suicide rates of those taking antidepressants compared to those who are not. Of those two-thirds, it is likely that many of them experienced symptoms of major depression but were not diagnosed and were untreated (84). These findings show the importance of screening for older adults’ depression as it can help physicians and healthcare professionals mitigate the risk of suicide before it is too late. A recent meta-analysis outlined the effectiveness of some SSRIs and selective norepinephrine reuptake inhibitors in decreasing older adults’ depression. Medications such as sertraline, paroxetine, and duloxetine decreased overall depression scores by 50% compared to the typical baseline for depressed older adults (86). This does not necessarily mean that all older adults given antidepressants will have significant reductions in their overall depression scores, as only about 50.7% of older adults respond to antidepressant treatment (83). Collectively, these findings are important and can be incorporated into other strategies to help reduce the risk of depression and suicide in older adults.

Exercise and physical activity have been proven to have beneficial impacts on older adults and their depressive symptoms (87). Older adults who frequently engage in physical activity for 150 minutes or more each week are significantly less likely to suffer from depression when compared to those who do not perform any physical activity (88). Beyond physical activity, regular flexibility exercises could also help reduce depressive symptoms. These positive effects occur due to improvements in both the physical and mental health of depressed older adults resulting in higher psychological well-being which ultimately reduces depression and lowers the risk of suicidal behaviors (89). Physical activity could also improve their physical strength and alter the levels of hormones in the body to ameliorate the effects of their depression (89). Exercise temporarily changes the levels of central norepinephrine, while decreasing the activity of the hypothalamopituitary-adrenocortical axis (88) and increasing the secretion of beta-endorphins (88, 89). These changes reduce feelings of depression in older adults and mitigate the risk of suicidal behaviors.

Depression and the risk for suicide are dependent on a variety of life stressors and are not just caused by one individual factor. Therefore, using several interventions and strategies to try and reduce the symptoms of depression and mitigate suicide risk is quite important. Combining the positive effects of exercise and physical activity with the effects of antidepressants has been shown to have greater improvements in mental health than using antidepressants alone (83). Using sertraline as an example, there were greater cognitive improvements when exercise and sertraline were used together rather than using sertraline alone. Each intervention has its benefits, and combining both leads to synergistic effects that reduce symptoms of depression in older adults (83). The study further suggested including exercise-based interventions in primary health care settings as these are the areas with relatively high concentrations of adults with late-life depression. Despite these findings, additional research is required to further understand the interactions of exercise and antidepressants as there are very few studies exploring these effects in the geriatric population.

### Suicide prevention

It is well-established that the general population is aging, placing a great deal of pressure on the provision of geriatric healthcare services, and contributing to the burden of late-life disease (90). Suicide among aging adults continues to receive less attention than it requires, and several factors can be considered when it comes to preventing suicidal thoughts and behaviors from a clinical standpoint.

Firstly, addressing the pervasive issue of ageism should be a universal priority; it is described as the negative attitudes towards aging that are held on both societal and individual levels (91). A 2020 systematic review with over 7 million participants on a global scale identified the fact that ageist practices and perspectives are related to poorer health outcomes in older adults worldwide, including increased mortality, slower recovery from illness, and mental health issues (92). In the context of individual healthcare settings, factors such as assumptions about poor cognition and functional decline can contribute to the failure to provide high-quality information during treatment, directly or indirectly leading to lower-quality care (92). As mentioned previously, social exclusion is a factor that contributes to suicidality in geriatric patients, and discrimination directed toward the population as a whole only further warrants intervention (93). Studies suggest that directed interventions such as intergenerational contact programs, and structured initiatives that allow for the development of meaningful relationships between individuals of different generations can help combat ageist perspectives and foster respect and admiration for those of advanced age (91). These interventions can be carried out via volunteering endeavours, school-based programming, recreational therapy in long-term care facilities, and community organizations that work with a variety of ages (94). Efforts such as these, in addition to media campaigns focused on positive portrayals of older adults, can help to directly address ageism, and foster mutual respect across the widening generation gaps in society (91).

Older adults as a whole are at risk for concerns that involve cognition, memory, and problem-solving; some of which are pathological, while others may be related to normal aging (95). Collectively, however, these changes are considered to be additional risk factors for the onset of suicidal ideation and behaviors (96). Some studies have demonstrated the efficacy of interventions that address cognition in older adults, such as problem-solving therapy (PST), a cognitive-behavioural technique which helps to minimize the negative impact of stress by helping patients approach and solve problems in a productive manner (97).

Lastly, there are some recommendations that clinicians can make to reduce the risk of harm by helping the patient manipulate their environment. Data from the USA suggests that firearms are involved in up to 70–80% of geriatric suicides, a substantial portion of which are in males (98). While Canada has more restrictions on firearm ownership, firearm-related suicide is still a concern, accounting for roughly 16% of all suicide fatalities in the country (99). Prevention strategies that help to manage the means aspect of suicide attempts include counselling patients on firearm safety like storage practices (such as the use of locking devices that prevent quick access, or community gun storage programs) and exploring the need for firearms through a clinical interviewing lens that may help patients to reconsider their ownership or licensure (98). Open and non-judgmental communication is key when approaching conversations about firearm safety, as gun ownership continues to be a value to some, especially those in rural communities (100).

### Screening for mental health disorders

Several different methods can be used to screen for depression in the geriatric population. Each has its advantages and disadvantages, but they are still effective at identifying the presence of depression. Diagnosing or identifying the risk of depression in the geriatric population is important for reducing the risk of suicide, as depression is one of the greatest risk factors for suicide in old age (28). A challenge to identifying depression in old age is the fact that depressed older adults are not as likely to show affective symptoms and are more likely to show somatic/vegetative symptoms and cognitive changes (101). These symptoms may be caused by physiological changes associated with aging, therefore decreasing the likelihood of detecting depression.

Two screening methods that are commonly used to assess depression in the geriatric population are the Geriatric Depression Scale (GDS) and the Depression in Old Age Scale (DIA-S). The GDS comes in various lengths, but the GDS-15 has only 15 questions and is frequently used in geriatric settings. The DIA-S has 10 items that are all answered in a yes or no format (102, 103). Important to both instruments are the positive predictive value (PPV) and the negative predictive value (NPV). Wunner et al. found that the GDS-15 had a greater PPV than the DIA-S, but the DIA-S had a greater NPV (2021). A greater PPV means that a patient with a positive screening value is more likely to have depression. A negative predictive value means that a patient with a negative screening value is more likely to not have depression which can be more accurate. Despite the greater PPV of the GDS-15, it has many questions that are generally considered unsuitable for geriatric patients (102). Some of the questions may lead to the acquisition of false data as they may answer yes on the GDS-15, but the actual reason for their answer is physical impairment or physiological changes associated with aging (102, 103). The DIA-S has questions that are typically more easily understood by patients in the geriatric population, and therefore require less clarification. Despite the differences between these two screening methods, however, there are still contrasting views on which screening method is the most suitable for depression screening. Some suggest that there is no clear preference for one screening instrument over the other (103) while others state that the DIA-S is a good alternative to the GDS-15 (102).

### Observing mood and behavioural changes in older adults

Identifying behaviors that signify a geriatric patient is at risk of suicide is crucial for timely intervention and support. Elderly individuals experiencing persistent sadness, hopelessness, a pervasive loss of interest in activities they once enjoyed, or expressing feelings of being a burden to others might be exhibiting warning signs of suicidal thoughts or actions (26).

### Suicide risk assessment

The first step when approaching a geriatric patient with concerns of depression or suicide in the clinical setting is to utilize a screening method for suicidal ideation (104).

The Geriatric Suicidal Ideation Scale (GSIS) is a 31-item scale that contains four subscales that assess suicide ideation, death ideation, loss of personal and social worth, and perceived meaning of life; this assessment scale and its pertinence to the subpopulation of geriatric patients have garnered support in the research community as having strong reliability and validity (105).

The 10-item Brief Geriatric Suicidal Ideation Scale (BGSIS) and the 5-item Geriatric Suicide Ideation Scale – Screen (GSISS) can be employed to identify and measure suicidal ideation in older adults (105). They are both targeted toward the geriatric population, thus helping to be inclusive in their ability to detect risk factors that may be more cohort-specific, including chronic health conditions, loss of loved ones, functional limitations, and loss of independence (104).

The Geriatric Depression Scale (GDS), which has both long and short versions, can also be used to detect elements of suicidality, but not necessarily stratify risk or imminence (106). Other scales that can be used during suicide risk assessment include the:

Columbia Suicide Severity Rating Scale (C-SSRS) can identify cases of actual suicide attempts and determine if there was a previous history of suicide attempts or non-suicidal self-injury (107).

The Suicide Intent Scale (SIS) measures suicide intent and has a total of 15 items (7 of them are subjective and 8 are objective) (108).

The suicide assessment scale (SUAS) measures symptoms known to be related to subsequent suicide irrespective of the diagnosis (109).

### Management of older adults who are suicidal

When older adults are at high risk for suicide, several interventions should be swiftly employed to provide support. The first step when presented with a suicidal geriatric patient is to establish an ongoing treatment plan; it is crucial to actively monitor and assess these patients closely, either in an inpatient or outpatient environment (79). While validated scales can be used to assess the severity of the patient’s suicidality, it is also encouraged to consider the factors that modify suicide risk. Depending on the content of the suicidal thoughts, there may be safety planning that should take place, and the clinician can routinely assess for access to firearms and other lethal means (110).

Multidisciplinary approaches to care are not only preferred but can be lifesaving in the context of geriatric suicide (110). While this article primarily focuses on the role of a psychiatrist or primary care provider, engaging other healthcare professionals including social workers, nurses, recreation therapists, and community outreach workers where applicable; considering the potential for hospital admission can be performed on an individualized basis (111). When it comes to physicians, studies have demonstrated that interdisciplinary care between geriatricians and psychiatrists with geriatric inpatients helps to reduce adverse outcomes such as polypharmacy (112).

If the patient is not admitted to the hospital, the clinician must provide recommendations for age-appropriate and accessible resources that can be accessed outside of standard business hours (111). This may include facilitating access to emergency departments, suicide hotlines, and first responders (113). Recognizing the influence that loneliness and isolation have on suicidal thinking, it would also be useful for mental health providers to equip themselves with the ability to facilitate interpersonal connections in their patients’ community – this can be accomplished through advocacy, liaising with family members, and familiarizing oneself with community programming (114).

The roles of psychotherapy, pharmacology, and electroconvulsive therapy (ECT) can be considered on an as-needed basis. While cognitive-behavioural therapy (CBT) is considered the first-line psychotherapeutic intervention for depression and suicidality in the general population, some special considerations need to be considered when treating older adults (115). Modification of psychotherapeutic techniques may need to be employed to accommodate for issues like cognitive decline, hearing loss, and unfamiliarity with technology (116). The medications that are preferred first-line agents, given their tolerability and efficacy, are duloxetine, mirtazapine, sertraline, venlafaxine, vortioxetine, citalopram, desvenlafaxine, and escitalopram, with an emphasis on frequent follow-up to optimize adherence (117). Failure to respond to first-line therapy warrants specialist referral or guideline-directed medical therapy for combination and/or augmentation strategies (118). Finally, ECT is a safe and effective treatment for depression in older adults, and it has even been shown that older adults who receive ECT have lower rates of mortality compared to alternative antidepressant therapies (119). ECT has evidence in reducing suicide risk (120). When a shorter duration to symptom resolution is prioritized, like in those at risk for suicide, ECT used in combination with venlafaxine can be a rapidly acting and effective treatment in depressed geriatric patients and has evidence in reducing suicide (121). Further research is required to better understand the safety and utility of other factors for treatment-resistant depression and suicidality in older adults, including transcranial magnetic stimulation (TMS) and ketamine (122). Nova Scotia has a new suicide risk assessment tool that incorporates risk and protective factors, documentation of risk levels, communications with other care providers, and management strategies (123).

### Suicidal behaviors in older adults

Identifying behaviors that signify a geriatric patient is at risk of suicide is crucial for timely intervention and support. One significant indicator, as previously mentioned, is the manifestation of depressive symptoms, which strongly correlate with suicide risk in older adults (22). Older adults experiencing persistent sadness, hopelessness, a pervasive loss of interest in activities they once enjoyed, or expressing feelings of being a burden to others might be exhibiting warning signs of suicidal thoughts or actions (26).

Changes in behaviour or mood can also be monitored, such as increased irritability, agitation, and anxiety potentially signifying an elevated risk. These alterations might be accompanied by disrupted sleep patterns, changes in appetite, or a decline in personal care (104). Studies have suggested that the presence of these behavioural changes when observed in conjunction with depressive symptoms, could serve as important indicators of potential suicidal tendencies among older adults. Moreover, explicit verbal cues or expressions of suicidal thoughts should not be dismissed; those expressing statements about wanting to die or expressing thoughts of being a burden to their loved ones, warrant immediate attention and intervention (111). It is important to emphasize the significance of taking such statements seriously and engaging in open conversations about suicidal ideation, ensuring the individual receives appropriate support and professional care (124).

By closely observing and recognizing these behavioural and verbal indicators in geriatric patients, caregivers, healthcare professionals, and family members can play a crucial role in identifying those at risk of suicide, providing timely support, and potentially preventing tragic outcomes.

## Discussion

Geriatric suicide remains a significant public health concern, as evidenced by the literature reviewed in this paper. The epidemiology of suicide among older adults reveals concerning trends, with some evident variation in demographic and socio-economic factors. The identification of risk factors for geriatric suicide, including psychiatric disorders, substance use, and social isolation, underscores the complex interplay of biological, psychological, and social determinants contributing to suicidal behaviour in this population. Despite advances in understanding the multifactorial nature of suicide in older adults, several discrepancies among researchers were noted, particularly regarding the role of specific risk factors and their relative importance in predicting suicidal behaviour. For instance, while psychiatric disorders, such as depression and anxiety, are widely recognized as significant contributors to suicidal behaviour among older adults, the extent to which these conditions independently increase the risk of suicide remains a topic of debate. Similarly, the role of substance use, social isolation, and physical health comorbidities in predicting suicidal behaviour in older adults varies across studies, highlighting the need for further research to clarify these discrepancies and establish consensus on the most salient risk factors for geriatric suicide. Future research should aim to clarify these issues to provide more robust evidence for the development of targeted prevention and intervention strategies.

One notable gap in the literature pertains to the impact of the COVID-19 pandemic on geriatric suicide rates and associated risk factors. While preliminary evidence suggests a potential increase in psychological distress and suicidal ideation among older adults during the pandemic, further research is needed to elucidate the long-term consequences and identify effective interventions to mitigate the adverse effects on mental health. Additionally, the role of protective factors against suicide, such as social support, spirituality, and access to mental health services warrants further investigation to inform comprehensive suicide prevention efforts tailored to the unique needs of older adults. A pertinent knowledge gap is the little knowledge about age group differences within the broad group of older adults such as age 60 to 70 versus 80 to 90. The role of medical problems in perpetuating suicide among different age groups remains unclear.

Despite the availability of evidence-based interventions for suicide prevention and management in clinical settings, challenges remain in implementing these strategies effectively. Limited resources, stigma surrounding mental health issues, and barriers to access to care pose significant obstacles to delivering timely and appropriate interventions for older adults at risk of suicide. Addressing these challenges requires a multifaceted approach involving collaboration between healthcare providers, policymakers, and community members to enhance awareness, improve screening and assessment protocols, and expand access to mental health services for older adults. Ultimately, by addressing the gaps in research and practice identified in this review, we can work towards reducing the burden of geriatric suicide and promoting the well-being of older adults worldwide.

## Conclusion

Despite the decrease in global suicide mortality rates, the rates of suicide in older adults continue to increase. With the anticipated growth in the size of the geriatric population, these numbers are only expected to continue growing if no further actions are taken. The complex etiology of geriatric suicide and the inability to easily detect suicide risk make it challenging for clinicians to intervene. Depression in older adults is commonly considered a symptom of aging and usually goes undiagnosed in older adults. To improve clinicians’ ability to detect and diagnose depression and subsequently lower the risk of suicide, it is important to understand other risk factors that could potentially signify suicidal behaviour. Besides MDD, which is one of the greatest risk factors for suicide, other factors like previous suicide attempts and alcohol consumption should be taken seriously. Additionally, after the COVID-19 pandemic and strict isolation procedures, the literature has revealed contrasting views on the impact of the pandemic and geriatric suicide risk. To further understand this relationship, we suggest that additional research be completed on this topic in the upcoming years. Moreover, we recommend that clinicians develop more evidence-based methods to identify the risk of suicide in geriatric patients to improve early suicide detection and prevention.

## Author contributions

JS: Writing – original draft, Writing – review & editing. BD-P: Writing – original draft, Writing – review & editing. LS: Writing – original draft, Writing – review & editing. LM: Writing – original draft, Writing – review & editing.
